# Willingness toward post-mortem body donation to science at a Mexican university: an exploratory survey

**DOI:** 10.1186/s12910-023-00982-1

**Published:** 2023-11-25

**Authors:** I. Meester, M. Polino Guajardo, A. C. Treviño Ramos, J. M. Solís-Soto, A. Rojas-Martinez

**Affiliations:** 1 Department of Basic Sciences, School of Medicine, Universidad de Monterrey, Av. Ignacio Morones Prieto 4500 Pte., Col. Jesús M. Garza, C.P, 66238 San Pedro Garza García, Nuevo León Mexico; 2 Instituto de Investigaciones en Bioética, Calzada del Valle 702, C.P, San Pedro Garza García, 66224 Mexico; 3 Department of Physiology, Faculty of Odontology, Universidad Autónoma de Nuevo León, Eduardo Aguirre y Pequeño s/n, C.P, Monterrey, 64460 Mexico; 4 Escuela de Medicina y Ciencias de la Salud and The Institute for Obesity Research, Tecnologico de Monterrey, Ave. Morones Prieto 3000, Col. Los Doctores, C.P, Monterrey, 64710 Mexico

**Keywords:** Bioethics, Biomedical research, Medical education, Post-mortem, Whole-body donation

## Abstract

**Background:**

Voluntary post-mortem donation to science (PDS) is the most appropriate source for body dissection in medical education and training, and highly useful for biomedical research. In Mexico, unclaimed bodies are no longer a legal source, but PDS is legally possible, although scarcely facilitated, and mostly ignored by the general population. Therefore, we aimed to evaluate the attitude and willingness for PDS and to identify a sociodemographic profile of people with willingness toward PDS.

**Methods:**

A validated on-line survey was distributed by the convenience method via the social networks of a Catholicism-inspired, private university in northern Mexico. Frequency analyses of all variables and coded free comments were complemented with association studies.

**Results:**

Although the responder cohort (*n* = 143) was too small and biased to be representative of the university community (*n* = 13,500), willingness to post-mortem organ donation was 90.7% and to PDS 70.7%. In this cohort, PDS willingness had the strongest association with mature age (> 40 years old; *P,* 0.0008). Among young adults, willingness to PDS was the lowest among volunteers from technical and business schools and the highest among those from the social sciences (*P*, 0.009). Respondents from the social sciences were also the most consistent between attitude and behavior with respect to organ donation. A free comment option revealed respondents were interested in the unusual taboo topic.

**Conclusions:**

A small, but sufficiently large proportion expressed willingness toward PDS. In our university cohort, which was biased in higher education and altruism, mature age and social interest were associated with PDS willingness.

**Supplementary Information:**

The online version contains supplementary material available at 10.1186/s12910-023-00982-1.

## Background

The use of human cadavers for biomedical education, training, and research has a long tradition in many countries. Although alternatives exist (*e.g*.*,* simulators, virtual reality, in vitro studies, and animal models), these should be considered complements rather than substitutes. Post-mortem bodies provide unique learning experiences, not only on the skills [1, 2] and critical thinking level [3], but also on emotional and ethical aspects of cadaver handling [4]. For research, whole-body donations are even more indispensable [5]. The use of alternatives seems to be guided more by feasibility than external validity or reliability [6]. The consequences of using more accessible but inappropriate animal models in research are severe in both ethical and economic aspects [7–9]. Although the use of bodies for educational purposes is declining in some countries [10, 11], its use in research is growing [10]. In contrast to post-mortem organ donation (POD), bodies from elderly persons or from deceased patients who had suffered infections or other diseases are valuable for scientific purposes, as they provide pathophysiological lessons or information to be discovered [12–14]. Thus, almost any deceased body could be valuable for medical education, training, and research [9].

The International Federation of Associations of Anatomists recommends that the ethical source of deceased bodies for scientific purposes is altruistic donation by individuals who express such destiny *ante mortem* [15]. Post-mortem donation to science (PDS) is a sensitive topic for many people, especially in strongly religious countries [16]. PDS is favored by i) a comprehensive legislation with opt-in and opt-out systems [16–19]; ii) a donor registry, as exists in Mexico for post-mortem organ donation (POD) [20]; iii) economic benefits or other benefits to society [17]; and iv) a well-educated population that is well-informed on the topic [21, 22], and extensive practical regulations and guidelines [23–29]. In studies among registered whole-body donors, informal communication between family and friends seemed important to raise awareness [21, 30, 31]. Internationally, countries in the Western world have a higher prevalence of whole-body donation than other countries. The impression is that religion, culture, and folk beliefs play a role in disposition [26, 32]. In countries without donation programs, bodies for research and teaching tend to be unclaimed bodies [2, 32].

In Mexico, previous legislation enabled both explicit PDS and the use of unclaimed bodies [33]. In practice, Mexican medical schools used to rely mainly on unclaimed bodies [32]. Body donation programs are scarce and have a short history. The first post-mortem body donation program started in 2016 at Mexico’s largest public university [34], which was followed, in 2019, by the largest public university in northern Mexico [35]*.* Mexican legislation prohibits the export of organs and cadavers outside the national territory, as well as any commercialization of cadavers or their parts [33]. Each individual possesses the primary right to determine the ultimate disposition of his or her cadaver. Individuals with secondary and tertiary rights are also specified. Disposition options include full or partial donation for transplantation, educational purposes, or research. Furthermore, individuals may specify particular circumstances or conditions [33].

In 2017, a new Mexican law, enacted to improve quests for missing people, ceased the supply of unclaimed bodies for education and science [36]. Many medical schools implemented simulators and virtual reality rather than body donation programs. The apparent disinterest in PDS among the medical community raised the hypothesis that non healthcare professionals would be more open to PDS programs than the medical community. A single Mexican survey on PDS willingness was limited to healthcare professionals and students [37]. Studies on post-mortem donation attitudes of the general Mexican population have been limited to POD [22, 38]. As information on the willingness to PDS of the general Mexican population is elusive, an online survey was carried out to verify the attitude toward PDS of an entire community of a private university in northern Mexico, as well as the proportion of individuals willing to PDS and to identify a sociodemographic profile of these individuals.

## Methods

### Design and participants

An observational, cross-sectional, anonymous, exploratory on-line survey was conducted among adults at a private Mexican university: the University of Monterrey. The university is within the metropolitan area of Mexico’s third largest city, Monterrey. The university is of Catholic inspiration, but open to all creeds and backgrounds, and attracts students from all over the country, although mainly from the northern states. People in northern Mexico tend to be more liberal with respect to business and technology, but more conservative on cultural topics than people in the central and southern regions of the country. The target population was the university’s community of about 13,500 adults, consisting of students (*n* = 12,588) and employees (*n* = 905) [39].

### Ethics

The institutional legal affairs office allowed the study to be conducted at its campus. Recruitment was started after the institutional Research and Ethics Committees had approved the protocol (*CEI-EM* 04–2021-02). After providing a web-based informed consent, volunteers completed the survey. Data collection was anonymous.

### Questionnaire

The questionnaire consisted of three sections: a 33-item section on attitudes and willingness toward post-mortem donation (PD) (Additional file 1), a 19-item sociodemographic section, and an open question to share any free comment.

The PD section was a modification of a validated Mexican questionnaire on attitudes toward POD [40], using a 5-point Likert scale (0–4, “Totally disagree” – “Totally agree”). Some POD items remained intact, while others were repeated or edited with a focus on PDS or to create an equilibrium between trust and distrust items. Thus, the modified PD section of the questionnaire covered three main aspects: POD, PDS, and trust; 11 items each: 5 favorable and 5 unfavorable attitude items plus one item on personal willingness.

The sociodemographic and socio-affective variables related to altruism and health can be inferred from Table 1.

**Table 1:** Sociodemographic profile and association with willingness to donate to science

Demographic variable	Total frequency, *n*(%)	PDS willingness frequency, n		*X*^2^ or Fisher test values	
		Negative	Positive	*X*^2^(df)	*P*
Highly homogeneous sociodemographic characteristics					
Nationality^a^					
Mexican	140 (97.9)	42	98	NA	0.555
Non-Mexican	3 (2.1)	0	3		
Region					
North	127 (88.8)	38	89		
Center	13 (9.1)	4	9	1.278(3)	0.734
South	2 (1.4)	0	2		
Non-Mexican	1 (0.7)	0	1		
Residence					
Urban	136 (95.1)	39	97	0.645(1)	0.422
Rural	7 (4.9)	3	4		
Social class					
Middle-income	122 (85.3)	34	88	0.995(2)	0.608
High income	15 (10.5)	6	9		
Low income	6 (4.2)	2	4		
Completed education level^a^					
High school +	142 (99.3)	41	101	NA	0.293
Basic obligatory	1 (0.7)	1	0		
Heterogeneous sociodemographic characteristics within the age cluster					
Age^a^					
< 40 years old	110 (76.9)	40	70	9.82(1)	0.0017
> 40 years old	33 (23.1)	2	31		
Civil status					
Single	96 (67.1)	36	60	ND	ND
Married	36 (25.2)	5	31		
Free Union	5 (3.5)	1	4		
Divorced	5 (3.5)	0	5		
Other	1 (0.7)	0	1		
Completed education level					
High school	72 (50.3)	30	42		
Postgraduate	59 (41.3)	8	51	ND	ND
Undergraduate	11 (7.7)	3	8		
Other	1 (0.7)	1	0		
University Role					
Student	78 (54.5)	32	46	ND	ND
Professor	43 (30.1)	6	37		
Other	22 (15.4)	4	18		
Non-age-related heterogeneous sociodemographic variables					
Gender					
Women	87 (60.8)	23	64	0.922(1)	0.337
Men	56 (39.2)	19	37		
Religion^b^					
Christian	110 (76.9)	37	73	NA	0.109
No religion	28 (19.6)	4	24		
Non-Christian religion	5 (3.5)	1	4		
Academic interest					
Health	51 (35.7)	12	39	14.178(4)	0.007
Social sci & Hum	32 (22.4)	4	28		
Business	24 (16.8)	12	12		
Technical	23 (16.1)	11	12		
Arts & Design	13 (9.1)	3	10		
Health-associated socio-affective characteristics					
Blood donor					
No	73 (51.0)	28	45	5.804(1)	0.016
Yes	70 (49.0)	14	56		
Registered organ donor					
No	79 (55.2)	29	50	4.582(1)	0.032
Yes	64 (44.8)	13	51		
Physical health					
Good	105 (73.4)	32	73	1.09(1)	0.297
Problems	38 (26.6)	10	28		
Mental health					
Good	96 (67.1)	26	70	0.44(1)	0.507
Problems	47 (32.9)	16	31		
Blood recipient					
No	134 (93.7)	39	95	0.073(1)	0.787
Yes	9 (6.3)	3	6		
Transplant, beloved ones					
No	132 (92.3)	38	94	0.281(1)	0.596
Yes	11 (7.7)	4	7		
Chronic disease patient					
No	131 (91.6)	40	91	1.019(1)	0.313
Yes	12 (8.4)	2	10		
Chronic disease, beloved					
No	79 (55.2)	24	55	0.087(1)	0.768
Yes	64 (44.8)	18	46		
PD willingness					
POD willingness^a^					
Yes	130 (90.9)	29	101	34.388(1)	0.000
No	13 (9.1)	13	0		
Trust willingness^a^					
Yes	87 (60.8)	23	64	0.577(1)	0.439
No	56 (39.2)	19	37		

^a^, Dichotomized data; in case of health status, minor and major problems were combined as there were very few major problems; in case of Likert score of Willingness: Yes includes “Totally agree” and “Agree”, and No includes “Totally disagree”, “Disagree” and “Neutral”). Abbreviations: *NA* not applicable, *ND* not done due to confounding by age, *PD* post-mortem, *PDS* PD to science, *POD* post-mortem organ donation

To ensure a questionnaire with correct and understandable Spanish and to estimate response time and reliability (Cronbach’s alpha, see below), the questionnaire was piloted among relatives, co-workers, and visitors of public neighborhood parks (*n* = 20). The pilot study revealed a response time rate of 8 to 15 minutes and a responder bias in favor of donation despite special efforts to include people with an unfavorable opinion; a phenomenon repeated in the main study.

### Recruitment

An e-card invitation with a hyperlink and a QR code was distributed via six institutional Facebook sites, an online news board, and supplementary directed e-mails and WhatsApp messages between September 19–22, 2021. Recruitment was stopped on September 27 when no new answers were received for 5 days in a row. The online survey was supported by QuestionPro and started with an informed consent that specified that any adult (≥ 18 years old) working or studying at the university campus of any opinion on the topic could participate. Incomplete questionnaires were not considered.

### Analyses

Categorical data were registered with numbered codes. To evaluate the internal consistency reliability of the PD section of the questionnaire, Cronbach’s α coefficient was determined with the online Wessa.net calculator [41] using the data of all valid responses.

Primary processing of the PD data included the calculation of total attitude scores per aspect as follows: $Attitude_{total}\;=\Sigma\;score\;favorable\;items\;-\;\Sigma\;score\;unfavorable\;items$.

The free comments were coded into categories (negative, neutral, and positive attitude) and subcategories according to arguments for the attitudes.

Frequency analysis was performed on all sociodemographic variables and on the coded categories from the free comments. Possible correlations were explored via a Spearman coefficient matrix with the following interpretation of ρ values: |ρ| > 2.0 relevant, but weak (±); |ρ| > 5.0 (+), strong; |ρ| > 7.0 very strong (++). Promising correlations were verified with Pearson Chi-Square (*X*^2^) or Fisher’s Exact tests, which are the valid tests for categorical data. Classes with recounts below 5 were joined when justifiable because of similar distributions. The significance level was set at *P* < 0.05 for all tests. Statistical tests were performed with SPSS v. 25, GraphPad Prism v. 9.2, and VassarStats.net [42].

## Results

### Small, self-selected responder cohort

Participants in the institutional target community (about 13,500 persons) reacted quickly to the on-line invitation, or not. In all, the mixed probabilistic and non-probabilistic convenience recruitment strategy yielded 733/13,500 visits to the survey (5.4% of the target population); 173/733 participants continued past the informed consent step (23.7% of visits), and 143/173 completed the questionnaire (82.7% of respondents). Thus, 1.1% (143/13,500) of the target population completed the survey.

### Sociodemographic characteristics and the relation to PDS willingness

**Fig. 1:**
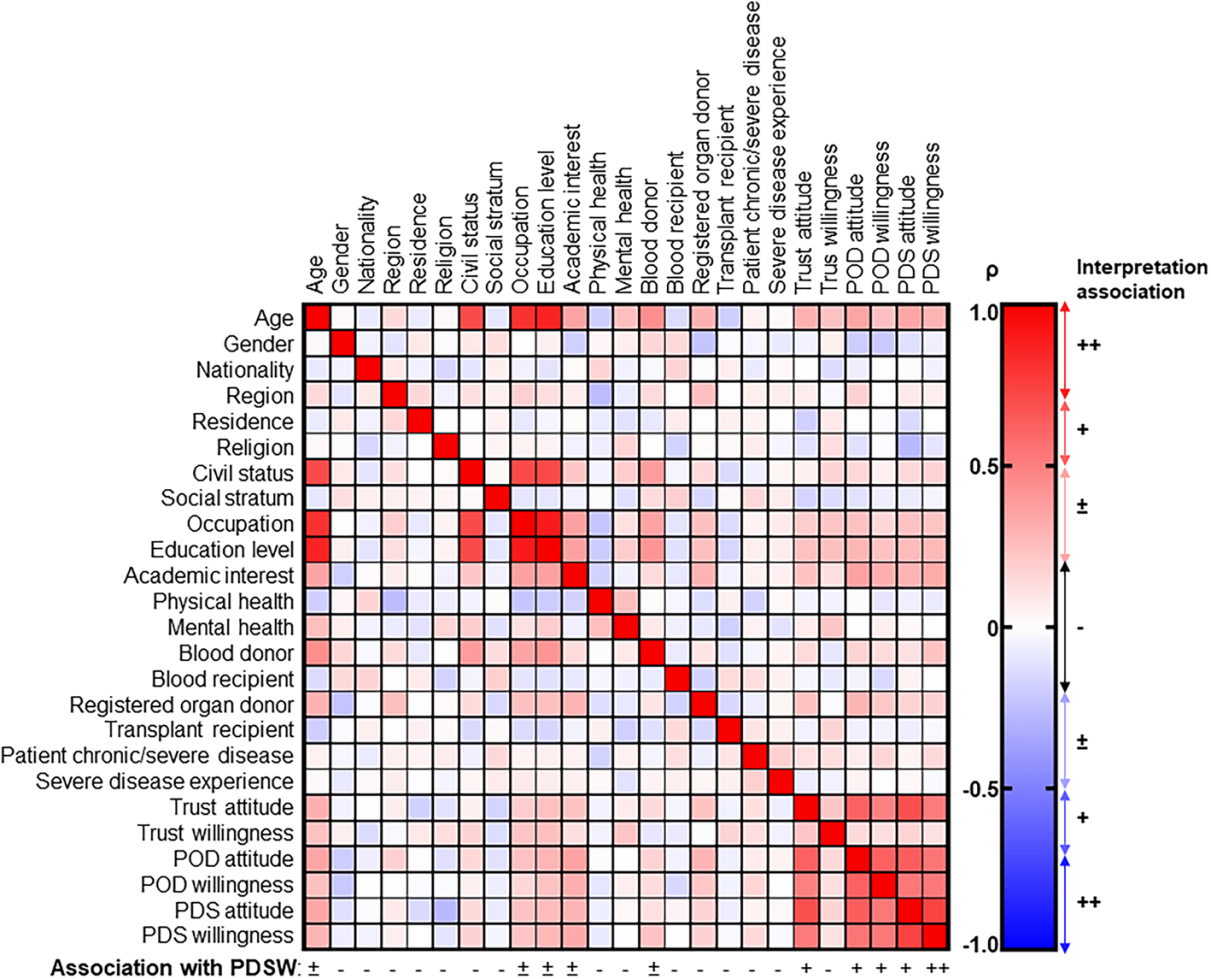
Explorative association among sociodemographics and post-mortem donation attitudes and willingness (*n* = 143). Abbreviations: POD, post-mortem organ donation, PDS, post-mortem donation to science; PDSW, PDS willingness

Table 1 presents the sociodemographic characteristics of the responder cohort. The responder cohort was highly homogeneous for nationality, region, residential area, family income, and education level (85.3–99.3%). Therefore, a potential impact of these variables on PDS willingness could not be detected. The age ranged from 18 to 67 years old, with a highly right-skewed distribution (+ 1.006), because the majority was 18–40 years old (young adults). Civil status, education level as of high school, and university role were well distributed over their respective classes (Table 1) but associated significantly and strongly among each other and with “Age” (|ρ|, 0.704–0.860; *P* < 1.0 × 10^−22^; Fig. 1); Fisher’s Exact with respect to Age: *P* < 0.0001 for all. As “Age” was considered a confounder for the other variables, the latter were not further analyzed in correlation studies. Gender, religion, and academic interest had distributions that seemed to reflect the campus population (Table 1). Neither gender nor religion was significantly associated with willingness to PDS, but academic interest was. Among the health-related socio-affective characteristics only “Blood donor” and “Registered organ donor” were significantly associated with PDS willingness. No association was found between PDS willingness and personal or indirect experiences with health issues. Most volunteers with health issues reported minor issues. Three responders reported major physical and/or mental issues. To enable appropriate association tests, volunteers with minor and major health issues were joined. Although patient organizations tend to promote, support, and facilitate medical education and research, no association was found between personal or indirect health issues and PDS willingness. Finally, POD willingness associated most significantly with PDS willingness (Table 1).

### Reliability and consistency of the PD part of the questionnaire

**Fig. 2:**
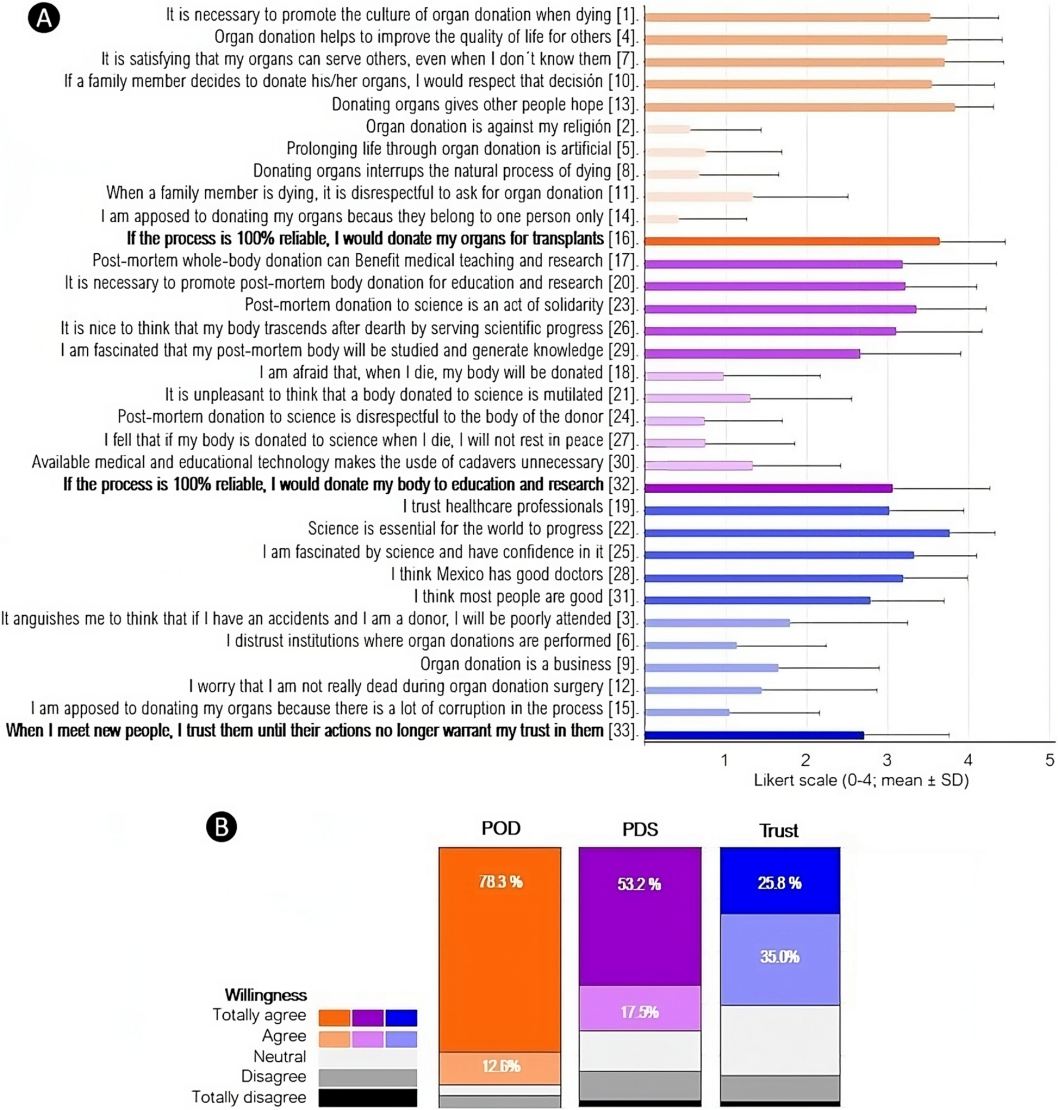
Post-mortem donation survey results. [#] item number within the PD questionnaire

The Cronbach’s α was 0.933 for the PD questionnaire, 0.913 for the PDS aspect, 0.845 for the POD aspect, and 0.801 for trust. Thus, the questionnaire as a whole and for each section yielded reliable data. As expected, favorable and unfavorable attitude items had a strong negative correlation for all aspects (ρ _PDS_, − 0.731; ρ _POD_, − 0.565, and ρ _Trust_, − 0.460; POD; *P* < 1.0 × 10^−8^ for all; Fig. 2A). As the scores on favorable items were higher than those on unfavorable items, the net attitude score was favorable for all aspects (Fig. 2A).

Attitude and personal willingness correlated significantly for all aspects with the following order based on the strength of correlation: ρ_PDS_, 0.737 (*P* < 1.0 × 10^−25^) > ρ_POD_, 0.617 (*P* < 1.0 × 10^−15^) > ρ_Trust_, 0.227 (*P* = 0.007) (Fig. 1). The rather low correlation between the trust scores for attitude and willingness revealed that a personal willingness to trust a new person was weakly associated with more general trust opinions. The net trust attitude score correlated more strongly with willingness to donate post-mortem in any format (ρ, 0.520 and *P* < 1.0 × 10^−10^ for PDS; ρ, 0.494 and *P* < 1.0 × 10^−9^ for POD) than the willingness to trust somebody new (ρ, 0.227; *P*, 0.007).

### A high proportion of the cohort is willing to donate post-mortem

A high proportion of the respondents were willing to donate their body post-mortem, especially POD willingness (*n* = 130, 90.9%), of which the majority were highly convinced (*n* = 112, 78.3%) (Fig. 2B). PDS willingness was a bit lower (*n* = 101, 70.7%), but more than half of these were still highly convinced (*n* = 76, 53.2%). In general, trust and POD attitudes correlated strongly with PDS attitudes and willingness: 0.520 ≤ ρ ≤ 0.690; *P* ≤ 2.7 × 10^−11^ (Fig. 1).

### Profile of respondents willing to donate to science

PDS willingness correlated significantly with the following variables: POD willingness > mature age (> 40 years old) > academic interest (social sciences and humanities, rather than business and technology) > being a blood donor > being a registered organ donor (Table 1). Age was also a confounder for the latter three variables because mature adults were more prevalent in the social sciences (*P*, 0.034), had more often been blood donors (*P* < 0.001), and were more often registered as organ donors (*P*, 0.0051) than young adults. After age correction, only the academic interest field maintained a significant association among young adults (*X*^2^(df), 9.482(2); *P*, 0.009) with the following order of PDS willingness frequency: social sciences and humanities > health and arts > business and technology. Thus, in our convenience responder cohort, PDS willingness was more common among respondents willing to POD, adults > 40 years old, and respondents with an interest in social sciences and humanities.

### Willingness and consistent behavior

As respondents with an academic interest in social sciences had the highest proportions of blood donors and registered organ donors (Table 2), the consistency between willingness and consistent behavior was analyzed. As none of the respondents was a registered PDS, consistency between willingness and behavior was verified among respondents willing to POD. Among 130 respondents willing to donate to POD, 64 (49.2%) were registered as such (Table 2). Age and academic interest had significant correlations with the self-reported POD registry. Mature adults were more consistent than young adults (*X*^2^(df) = 8.33(1)*; P,* 0.0039; Table S1, Additional file 2), and young adults with an academic interest in the social sciences were more consistent than those of other academic interests (*X*^2^(df) = 11.98(4)*; P,* 0.018; Table S2, Additional file 2). Respondents of the social sciences also had the highest proportion of blood donors, which was interpreted as altruistic behavior (Table 2). Thus, in this cohort, volunteers from social sciences combined a relatively high level of altruism and willingness to donate post-mortem with a high level of consistent self-reported behavior.

**Table 2:** Willingness and behavior to donate blood and post-mortem organs according to academic interest

Academic interest	*n*	Blood donor, *n* (%)	Willingness POD, *n* (%)	Registered POD, *n* (%)	Consistency Willingness - Registry, *n* (%)
Business	24	11 (45.8)	20 (83.3)	4 (16.7)^a^	4/20 (20.0)^c^
Technical	23	10 (43.5)	17 (73.9)	7 (30.4)	7/17 (41.2)
Health	51	19 (37.3)	51 (100)	26 (51.0)	26/51 (51.0)
Arts & Design	13	6 (46.2)	12 (92.3)	6 (46.2)	6/12 (50.0)
Social sciences & Humanities	32	24 (75.0)^a^	30 (93.8)	21 (65.6)^b^	21/30 (70.0)^c^
Total cohort	143	70 (49.0)	130 (90.9)	64 (44.8)	64/130 (49.2)

^a^, *X*^*2*^(4) = 11.891, *P* = 0.018, significant; ^b^, *X*^*2*^(4) = 16.013, *P* = 0.03, significant; ^c^, *X*^*2*^(4) = 12.52, *P* = 0.014, significant; *POD* post-mortem organ donation

### Free comments

Only 15 out of 143 respondents (10.5%) took advantage of the opportunity of the free comment option. Thirteen comments were positive, one was neutral, and one was negative (Table 3). The single negative comment raised the issue of whether scientific progress was a valid cause for PD. The neutral comment came from a participant who wanted to know the results of the study, which were communicated via an infographic through the same communication channels used for recruitment. Positive comments revealed emotional, social, and utilitarian motivations, expressed through phrases such as “interesting”, “increasing awareness¨, “normalizing a taboo”, and “useful”. Only positive commentators were willing to PDS. Positive commentators with emotional motives tended to have higher trust scores than those with utilitarian or social awareness motivation.

**Table 3:** Free comments from respondents of the PD questionnaire

Comment type	Qualifier (class)	Frequency commentator characteristics (*n*)			
		All	Willingness		
			Trust	POD	PDS
Negative	Science no good cause (Rational)	1	0	1	0
Neutral	Information (Rational)	1	1	0	0
Positive	PD favorable (Mix)	13	7	13	11
	Interesting (Affective)	7	7	7	6
	Awareness (Social)	6	2	6	5
	Useful (Rational)	3	1	3	3
Total	–	15	8	14	11

*PD *post-mortem donation, *PDS* PD to science, *POD* post-mortem organ donation

## Discussion

Most studies on willingness to PDS are limited to health professionals or students [43–46], although some reports have focused on other populations, such as blood donors [47], registered body donors [30], ethnicities [48], patients, and relatives [49]. With respect to Mexico, as far as we know, there is only one PDS willingness study, which was limited to the staff and students of an anatomy department [37]. The hypothesis that a small proportion of the northern Mexican population is willing to donate their post-mortem body to science and that non-health-related persons might be more willing than health-related professionals seemed accurate for a cohort recruited online from a private university community.

### Proportion willing to PDS

A promising result was that 70.7% of the responder cohort was willing to PDS. Another Mexican study on PDS willingness reported a similar proportion [37]. This latter cohort differed from ours in population and recruitment strategy. Rather than a study population limited to students and staff of an anatomy department, our population included students and personnel from an entire university community. With respect to recruitment strategy, rather than an invitation in a working or study environment, our online convenience strategy presented limited control and less social pressure for the study population. The convenience recruitment strategy seemed to yield a self-selected cohort with a double bias, altruism, and interest in the topic, which is discussed further in the profile section.

The proportion of our cohort willing to PDS represented 0.78% of the target population. A relatively high number in comparison to 0.17% of the Mexican population registered as organ donors at the federal transplant registry [20, 50]. It is also high in comparison to a progressive society, such as the Dutch, where 0.1% of the population was registered as a body donor in 2013 [51]. The comparison of survey with registry data is awkward because of the well-known discrepancy between willingness and behavior [21]. Two relevant aspects of these low proportions are the following: 1. The proportions tend to below the significance threshold, indicating that PDS willing people are significantly different from the general population. Most countries and cultures have a small, distinctive cohort willing to PDS [51]; 2. Low proportions tend to be sufficient; high proportions may generate an undesirable surplus of bodies [51]. Thus, a willingness rate of 0.78% for the university population may seem small but may be sufficient for successful PDS programs, especially if it also occurs at a national level.

### Sociodemographic profile of people willing to PDS

With respect to the profile of respondents willing to PDS, age had the strongest impact in our well-educated cohort at a private university. Mature adults were more willing toward PDS than younger adults. A similar age effect was reported from a PDS survey among staff and students from an anatomy department at a public university in northern Mexico [37] and is consistent with most international data [52, 53]. In contrast, a POD survey among the general population in central Mexico found that older participants had a less favorable attitude [54]. In the latter study, lower levels of education among older people may have been a confounding factor. Indeed, less education has been associated with more misconceptions, more psychological barriers, and less willingness [22]. Education at high school level or beyond is an important factor for a positive attitude toward PDS [31, 52]. The importance of age and education has been reported repeatedly in a variety of cultures [29, 31, 52–55], including Mexico [54]. As the education level of our cohort was relatively high, the impact of lower education was not evident. Hence, in our cohort, mature age was the most distinctive sociodemographic trait among people willing to PDS.

Among the young adult respondents (up to 40 years old), academic interest had a strong correlation with PDS willingness. Those with an academic interest in the humanities and social sciences were the most willing, while those interested in technology and business were the least willing to PDS. In contrast, a survey among Indian registered body donors found that engineers and businesspeople were more abundant than donors from the humanities and social sciences [31]. These contrasting findings may in part be due to cultural differences. The relatively low rate of PD behavior among medical physicians across different countries and cultures is notable [31, 52, 56]. Willingness to self-donate tends to decline after dissection experiences, while a positive attitude toward PDS by strangers remains intact [57–59]. This phenomenon was not found in the single study on PDS willingness among Mexican anatomy students [37]. However, an aversion due to dissection experience could explain why our respondents from the health sciences did not have the highest PDS willingness rate. In our cohort, all respondents from health sciences were in favor of POD, but only 50% reported being registered as such. Respondents from the social sciences had the highest consistency rate, with 70% reporting being registered as POD. The relationship between career choice, PD willingness, and consistent behavior is complex and beyond the scope of this study. To summarize, our university cohort showed a higher willingness to PDS among respondents from the humanities and social sciences, who also had the highest rate of self-reported consistent behavior toward PD.

### Health-related socioaffective characteristics

Socioaffective characteristics, such as social responsibility, benevolence, altruism, empathy, social responsibility, and trust have been reported worldwide as motivators for blood donation, POD, and PDS [60–62], including for a Mexican POD study [38]. Our cohort appeared to have an altruistic bias. An unexpectedly large proportion of respondents had previously donated blood (49.0%) and/or self-reported being a registered POD (44.8%). Although there are no reference data available for the target population, there is circumstantial evidence. Mexico is known for low rates of altruistic blood donation [63], and this also applies to Nuevo León [64], the state where most of the respondents came from. Although this study did not distinguish between altruistic and family-motivated blood donation, the relatively high proportion of blood donors in the respondent cohort suggests an altruistic bias. The proportion of registered organ donors in our study (44.8%) was higher than that reported in a Mexican POD survey among nursing and medical students (11–35%) from public and private universities in central Mexico [65]. In contrast to this latter study, where the POD registry was supported by physical evidence, our anonymous online study relied on self-reports. Although there was no social pressure in our study, over-reporting of actions considered socially desirable cannot be ruled out. Our recruitment method may have favored the self-selection of a cohort with an interest in the topic and an altruistic bias. The POD registry proportions are much higher than data from the federal POD registry (0.17%) [20, 50], probably due to their higher accessibility as they are linked to the issuance of a driver’s license. Altogether, our self-selected cohort seems to present an altruistic bias, which may explain the high proportion willing to PDS.

### Sociopsychological and cultural aspects

In our cohort, the proportion with willingness to POD (90.9%) was higher than that with willingness to PDS (70.7%). This is a common finding [58, 59, 66]. What determines these differences? People may imagine a greater disfigurement of the post-mortem body when it is destined for PDS than for POD. Mutilation of the post-mortem body, fear, and family considerations are strong contributors to POD and PDS aversion [57–59, 66], also in Mexico [38]. People may think that saving a life-saving POD is a better cause than PDS. Indeed, the utilitarian motive has been recognized for general PD willingness, including in Mexico [21, 38, 67]. In the free comments section of our survey, post-mortem usefulness was mentioned in a positive sense. However, for one POD-positive respondent, the uselessness of science was an argument against PDS, which still underscores the importance of the utilitarian motive. The most common positive terms in the comments were “interesting” and “social awareness”. Social awareness and interest are helpful first steps toward body donation as they motivate a search for information [21]. Importantly, the willingness rate tends to be higher than the rate of compliant behavior, as we noticed in the compliance of POD donors. The willingness-behavior discrepancy is not limited to PD but has been observed in many areas [68, 69]. A profound sociopsychological analysis of this phenomenon, although interesting, goes beyond the aims of this study.

With respect to cultural aspects, Mexico is portrayed for its idiosyncratic, ludic feelings toward death as an entity. The stereotype of Mexican death cults is accurate as an identity marker, but inaccurate because it is a one-sided exaggeration that fails to describe the full range of emotions that every human being experiences when confronted by death. Indeed, few Mexicans display ludic stoicism toward their own death and illness [70]. As in most countries and cultures, Mexicans vary not only individually but also by class, ethnicity, and region. As in most countries, in Mexico there is a minority willing to PD. A worldwide profile can be summarized as follows: PDS-willing people are a minority characterized by the following motivators: altruism and usefulness which seem to increase with age and education. On the other hand, fear, mutilation, and family considerations are demotivators. In general, Western world cultures have a higher prevalence of PDS willing people, but willingness to PDS exists in a minority in almost all cultures.

### Limitations

Valid responses represented only 1.1% of the target population. Web-based recruitment may not have reached the target population completely. Additionally, the tendency to not participate when holding a negative attitude towards PDS may explain the low participation rate. Due to the low response rate, the results are not representative of the target population and only describe the responder cohort. Recruitment difficulties for a PD survey have been reported previously [71]. Additionally, there is probably a nonresponse error, as 76.7% of visitors to the survey site did not proceed beyond the informed consent. This group was likely interested in the topic but discouraged for unknown reasons at the first step. Reasons for discouragement could be: i) the length or content of the informed consent, and ii) the time investment required, among others. Furthermore, 17.3% dropped out before completing the questionnaire. These dropouts may have been due to technical reasons, the length of the questionnaire, being disgraced by certain items, or other reasons. In the study design and during the pilot study, it was determined and verified that the questionnaire could be completed within 15 minutes. This is important, as it is known that data quality declines with longer surveys [72]. Furthermore, it is probable that the responder cohort had a sampling bias, with community members who were less attentive to the institutional sites and news board being underrepresented. Moreover, as mentioned earlier, an altruistic bias was perceived in the responder cohort. An incentive might have diminished this sampling bias. The altruistic bias may be smaller than it seems in case blood donation and POD registry were over-reported, as they may have been perceived as desirable answers. Overall, convenience recruitment and online surveys generate several reliability issues that are common in online surveys [73]. Because of recruitment issues, the 70.2% PDS willingness cannot be extrapolated to the target population, and can be extrapolated even less to the Mexican population. However, the existence of this nonrepresentative, small (0.78%), altruism-biased, PDS-willing group is relevant and promising as it may be extrapolatable to the Mexican population. Future studies will verify that.

## Conclusions

A small, but sufficiently large proportion expressed willingness toward PDS. With respect to the profile of people willing to PDS in our university cohort, which was biased in education and altruism, mature age was the most important factor and a social interest seemed beneficial. The study results are promising for organizing social awareness, education, and registries for PDS, so that deceased human bodies return to Mexican medical schools and research institutes, but this time in an ethically appropriate way. Trust, altruism, social interest, and mature age seem to be the main factors that convert a positive attitude into personal willingness to donate the post-mortem body to science.

### Supplementary Information

**Additional file 1.** PD questionnaire.pdf contains the questionnaire in both Mexican Spanish (original) and the English translation.

**Additional file 2.** ROD Age Academic-interest.pdf contains Tables S1 and S2, contingency table that on registered organ donation (ROD) according to age and academic interest among young adults.

**Additional file 3.** Survey results.xlsx contains the crude survey results. The Excel file contains the following 7 sheets: i) Code, ii) Sociodemographic, iii) PD data, iv) PD-Cronbach, v) PDS-Cronbach, vi) POD-Cronbach, vii) Trust-Cronbach.

## Data Availability

The dataset supporting the conclusions of this article is included within this article (Additional file 3).

## Abbreviations

PD: Post-mortem donation
PDS: Post-mortem donation to science
POD: Post-mortem organ donation
