# Supplementary material for: Willingness toward post-mortem body donation to science at a Mexican university: an exploratory survey
Source: BMC Med Ethics. 2023 Nov 25;24:101. doi: 10.1186/s12910-023-00982-1 (PMC10675933; doi:10.1186/s12910-023-00982-1)
Supplement: Supplementary file 1 — Additional file 1. PD questionnaire.pdf contains the questionnaire in both Mexican Spanish (original) and the English translation. [file 12910_2023_982_MOESM1_ESM.pdf]

PD questionnaire in two formats:

1. The original Spanish version for the Mexican population. The questionnaire was a modified version of a previously reported and validated questionnaire (Jasso et al., 2018).
2. The English translation of the original Spanish version. Items are grouped according to aspect.

1. **The original Mexican Spanish version** (Likert scale 0-4; 0, Totally disagree, 4, Totally agree)

Item #	Question original version	Aspect	Type
1	Es necesario fomentar la cultura de donación de órganos al morir.	POD	A
2	La donación de órganos va en contra de mi religión.	POD	A
3	Me angustia pensar que, si tengo un accidente y soy donador, sea mal atendido.	Trust	A
4	El donar órganos ayuda a mejorar la calidad de vida de otros.	POD	A
5	Prolongar la vida por medio de la donación de órganos es artificial.	POD	A
6	Desconfío de las instituciones en las que se realizan trasplantes de órganos.	Trust	A
7	Es satisfactorio que los órganos pueden servir a otros, aunque sean desconocidos.	POD	A
8	Donar órganos interrumpe el proceso natural de morir.	POD	A
9	Con la donación de órganos se hace negocio.	Trust	A
10	Si un familiar antes de morir decide donar sus órganos, yo haría lo necesario para que se lleva a cabo la donación.	POD	A
11	Cuando se está triste por la muerte de un familiar, es una falta de respeto pedir que sus órganos se donen.	POD	A
12	Me preocupa no estar realmente muerto cuando se haga la cirugía para la donación de órganos.	Trust	A
13	Al donar órganos se da esperanza a otras personas.	POD	A
14	Me opongo a donar mis órganos porque estos pertenecen a una sola persona.	POD	A
15	Me opongo a donar mis órganos al morir porque hay mucha corrupción en el proceso.	Trust	A
16	Si es proceso es 100% confiable, donaría mis órganos para trasplantes.	POD	W
17	Un future medico puede aprender a través de mi cuerpo muerto.	PDS	A
18	Me da miedo que al morir se done mi cuerpo.	PDS	A
19	Confío en los profesionales de salud.	Trust	A
20	Es necesario fomentar la donación del cuerpo postmortem a favor de la enseñanza e investigación.	PDS	A
21	Es desagradable pensar que el cuerpo donado a la ciencia y enseñanza médica queda mutilado.	PDS	A
22	La ciencia es fundamental para que el mundo avance.	Trust	A
23	El que alguien dona su cuerpo postmortem a la ciencia es una acción solidaria.	PDS	A
24	La donación del cuerpo postmortem a la ciencia es una falta de respeto para el cuerpo del que dona.	PDS	A
25	Me fascina la ciencia y tengo confianza en ella.	Trust	A

26	Es lindo pensar que mi cuerpo trasciende después de mi muerte al servir en el progreso científico.	PDS	A
27	Siento que si mi cuerpo se dona a la ciencia cuando muera no descansaría en paz.	PDS	A
28	Pienso que México tiene buenos médicos	Trust	A
29	Me fascina la idea que mi cuerpo postmortem será estudiado y generará conocimiento.	PDS	A
30	La tecnología médica y educativa disponible hace innecesaria el uso de cadáveres.	PDS	A
31	Creo que la mayoría de las personas es buena.	Trust	A
32	Si el proceso es 100% confiable, donaría mi cuerpo para la educación e investigación.	PDS	W
33	Al conocer nuevas personas, confío en ellas hasta que sus acciones ya no ameritan mi confianza.	Trust	W

A, Attitude; W, willingness; PDS, post-mortem donation to science POD, post-mortem organ donation;

2. English translation of the PD questionnaire with the items clustered according to aspect.

**Post-mortem organ donation (POD)**	**Item nr.**
Positive	
It is necessary to promote the culture of organ donation when dying.	1
Organ donation helps to improve the quality of life for others.	4
It is satisfying that my organs can serve others, even when I don´t know them.	7
If a family member decides to donate his/her organs before they die, I would do whatever is necessary for the donation to take place.	10
Donating organs gives other people hope.	13
Negative	
Organ donation is against my religion.	2
Prolonging life through organ donation is artificial.	5
Donating organs interrupts the natural process of dying.	8
When sad because of a dying family member, it is disrespectful to ask his/her organs to be donated.	11
I am apposed to donating my organs because they belong to one person only.	14
Attitude	
If the process is 100% reliable, I would donate my organs for transplants	16
**Post-mortem donation to science (PDS)**	
Positive	
Post-mortem whole-body donation can benefit medical teaching and research.	17

It is necessary to promote post-mortem body donation for education and research.	20
Post-mortem donation to science is an act of solidarity.	23
It is nice to think that my body transcends after death by serving scientific progress.	26
I am fascinated that my post-mortem body will be studied and generate knowledge.	29
Negative	
I am afraid that, when I die, my body will be donated.	18
It is unpleasant to think that a body donated to science and medical education is mutilated.	21
Post-mortem donation to science is disrespectful to the body of the donor.	24
I feel that if my body is donated to science when I die, I will not rest in peace.	27
Available medical and educational technology makes the use of cadavers unnecessary.	30
Attitude	
If the process is 100% reliable, I would donate my body to education and science	32
**Trust**	
Positive	
I trust healthcare professionals.	19
Science is essential for the world to progress.	22
I am fascinated by science and have confidence in it.	25
I think Mexico has good doctors.	28
I think most people are good.	31
Negative	
It anguishes me to think that if I have an accident and I am a donor I will be poorly attended.	3
I distrust institutions where organ donations are performed.	6
Organ donation is a business.	9
I worry that I am not really dead during organ donation surgery.	12
I am opposed to donating my organs because there is a lot of corruption in the process	15
Attitude	
When I meet new people, I trust them until their actions no longer warrant my trust in them.	33

Note: 5-scale Likert (0, totally disagree; 4, totally agree).
