# Supplementary material for: Willingness toward post-mortem body donation to science at a Mexican university: an exploratory survey
Source: BMC Med Ethics. 2023 Nov 25;24:101. doi: 10.1186/s12910-023-00982-1 (PMC10675933; doi:10.1186/s12910-023-00982-1)
Supplement: Supplementary file 2 — Additional file 2. ROD Age Academic-interest.pdf contains Tables S1 and S2, contingency table that on registered organ donation (ROD) according to age and academic interest among young adults. [file 12910_2023_982_MOESM2_ESM.pdf]

Table S1. Registered organ donation according to age.

Age (years old)	Non-registered POD, n (%)	Registered POD, n (%)	Total, n (%)
≤ 40	68	42	110
> 40	11	22	33
Total cohort	79	64	143

$X^2(1)$: 8.33, P = 0 0.0039, significant; POD, post-mortem organ donation.

Table S2. Registered organ donation according to academic interest among young adults.

Academic interest	Non-registered POD, n (%)	Registered POD, n (%)	Total
Business	19	2	21
Technical	13	6	19
Health	24	19	43
Arts & Design	5	5	10
Social Sciences & Humanities	7	10	17
Total cohort	68	42	110

$X^2(4)$ = 11.98, P = 0.018, significant; POD, post-mortem organ donation.
